# Intra- and Interpopulation Diversity of the Phytopathogenic Fungi of the *Microdochium nivale* Species

**DOI:** 10.3390/jof10120841

**Published:** 2024-12-05

**Authors:** Olga A. Gogoleva, Egor A. Ryazanov, Guzalia Sh. Murzagulova, Sergey N. Ponomarev, Inna B. Chastukhina, Ildar T. Sakhabutdinov, Elena V. Osipova, Gulnaz S. Mannapova, Viktor N. Korzun, Mira L. Ponomareva, Vladimir Yu. Gorshkov

**Affiliations:** 1 Federal Research Center “Kazan Scientific Center of the Russian Academy of Sciences”, 420111 Kazan, Russia; gogolewaoa@yandex.ru (O.A.G.); eg.ryazanov@gmail.com (E.A.R.); innachast@yandex.ru (I.B.C.); mgs1980@mail.ru (G.S.M.);; 2 Faculty of Natural Sciences, Novosibirsk State University, 630090 Novosibirsk, Russia; 3 KWS SAAT SE & Co. KGaA, Grimsehlstr. 31, 37555 Einbeck, Germany; 4 Institute of Fundamental Medicine and Biology, Kazan Federal University, 420008 Kazan, Russia

**Keywords:** intra- and interpopulation diversity of phytopathogenic fungi, *Microdochium nivale*, winter cereal crops, virulence, plant infectious diseases

## Abstract

Our study investigates the intra- and interpopulation diversity of phytopathogenic fungi, using *Microdochium nivale* as a model organism. A total of 136 strains, representing two populations, were collected from different winter cereal crops (rye, wheat, and triticale) across two agrocenoses. In these strains, we analyzed and compared genetic and phenotypic traits, exploring potential relationships between them. Significant genetic and phenotypic diversity was revealed among the assayed strains, with most exhibiting distinctive genetic features compared to previously characterized strains from other countries. Both populations included highly virulent and lowly virulent strains, with the majority displaying similar virulence levels across different cereal crops. Some strains demonstrated differential virulence toward different crops, and some even exhibited a plant-beneficial effect on rye while being virulent to wheat and triticale. The percentages of strains with specific genetic traits were different in the two analyzed populations. Our study lays the groundwork for identifying genetic markers associated with phenotypic traits in the studied strains, which will aid in developing reliable diagnostics for predicting crop loss risk and improving disease management. Furthermore, it will assist in identifying reliable sources of resistance to *M. nivale*-induced diseases within the winter cereal gene pool.

## 1. Introduction

Fungal species, including phytopathogenic ones, comprise genetically and phenotypically distinct forms. Geographical origin can contribute to intraspecific differences in genetic and phenotypic traits, with strains from distant regions exhibiting unique “geographic” characteristics [1,2]. For example, in Eurasia, at least three populations of *Fusarium graminearum* have been distinguished based on genetic markers associated with the production of different types of trichothecene mycotoxins, namely 3-acetyldeoxynivalenol (3ADON) and 15-acetyldeoxynivalenol (15ADON) [3]. Most Chinese populations of the strawberry root rot pathogen *Fusarium commune* have been found to possess unique alleles, indicating interpopulation diversity within this species [4]. At the same time, the intrapopulation diversity of *F. commune* has made a significantly greater contribution to the intraspecific diversity of this species than interpopulation diversity [4], and such a trend has been observed in the majority of phytopathogenic fungal species [1,5,6,7,8,9,10,11,12,13]. Even within a single field or plot, the intraspecific genetic diversity of fungi can be remarkably high, while local populations from distant regions share a considerable amount of genetic commonality.

Relatively little research has been conducted on how intrapopulation genetic diversity translates into phenotypic diversity [12,13,14,15,16]. Nevertheless, studies have consistently shown that different strains within a local population can exhibit varying levels of virulence and resistance to fungicides [11,14,15,17,18], which is a crucial consideration in agricultural practice. Consequently, investigating the genetic and phenotypic population diversity of phytopathogenic fungi is of great importance. The findings of these studies should be further applied to disease management strategies, particularly to distinguish between genotypes that pose a threat to crops (or a specific crop) and those that are less harmful or even plant-beneficial, within the framework of diagnostic measures.

High intraspecific and intrapopulation diversity is also a characteristic feature of the fungi within the *Microdochium nivale* species, which are responsible for various plant diseases [19]. *Microdochium nivale* is particularly notable as the causative agent of pink snow mold affecting winter cereals and grasses [20,21]. Snow mold is predominantly found in the Northern Hemisphere, including Europe, Japan, the United States, and Canada [22,23]. In central European Russia, snow mold often exceeds epiphytotic levels, leading to significant losses in winter crops [21,24,25,26,27]. In Canada, snow mold control accounts for approximately 50% of annual fungicide use on turfgrass [22]. Management of snow mold is particularly challenging due to the lack of resistant plant cultivars, limited donors of quantitative resistance for breeding programs, difficulties in applying fungicides under snow cover, and limited knowledge of the pathogens causing the disease [21]. Although snow mold was previously thought to be restricted to areas with prolonged snowy winters [28,29,30], *M. nivale*, the causal agent of pink snow mold, has adapted to warmer temperatures and shorter winters, enabling its spread into less snowy regions of Western and Eastern Europe [21,31,32,33]. Furthermore, *M. nivale* can cause not only snow mold but also seedling blight, brown foot rot, and head blight, with symptoms resembling those of Fusarium head blight [19,23,30,31,33,34,35,36,37,38,39,40,41,42,43,44,45] throughout the growing season.

The high intrapopulation genetic diversity of *M. nivale* has been clearly demonstrated in strains colonizing turfgrasses [46]. Each of the 100 strains analyzed in that study represented a distinct genotype, with intrapopulation variability contributing more significantly to intraspecific variability than interpopulation variability [46]. However, it remained unclear whether this high genetic variability was reflected in phenotypic variability. Both genetic and phenotypic intraspecific diversity have been demonstrated in 44 *M. nivale* strains isolated from grasses and cereals in Norway between 1975 and 2010 [47]. Clustering of these strains based on β-tubulin gene sequences corresponded to the host plants they were isolated from (grasses or cereals), and *M. nivale* strains originally isolated from grasses exhibited higher virulence on grasses (*Lolium perenne* and *Festulolium hybrids*) compared to strains from cereals [47].

To date, no studies have comprehensively examined both genetic and phenotypic intrapopulation and interpopulation diversity of *M. nivale* strains originating from cereal crops. In our previous study, we investigated the phenotypic intrapopulation diversity of *M. nivale* strains isolated from a particular crop, winter rye [48]. Although that study was limited to a relatively small sample of strains from a single agrocenosis, it revealed that *M. nivale* strains colonizing rye exhibit diverse phenotypes. The present study aims to assess the level of genetic and phenotypic intrapopulation and interpopulation diversity of *M. nivale* strains originating from different cereal crops and different agrocenoses. Specifically, we sought to answer the following questions: (i) What is the extent of genetic and phenotypic diversity within and between *M. nivale* populations from cereals? (ii) Do strains from different hosts or agrocenoses exhibit distinct genetic and phenotypic traits? (iii) Are there relationships between the analyzed genetic and phenotypic traits?

## 2. Materials and Methods

### 2.1. Sample Collection

**Figure 1:**
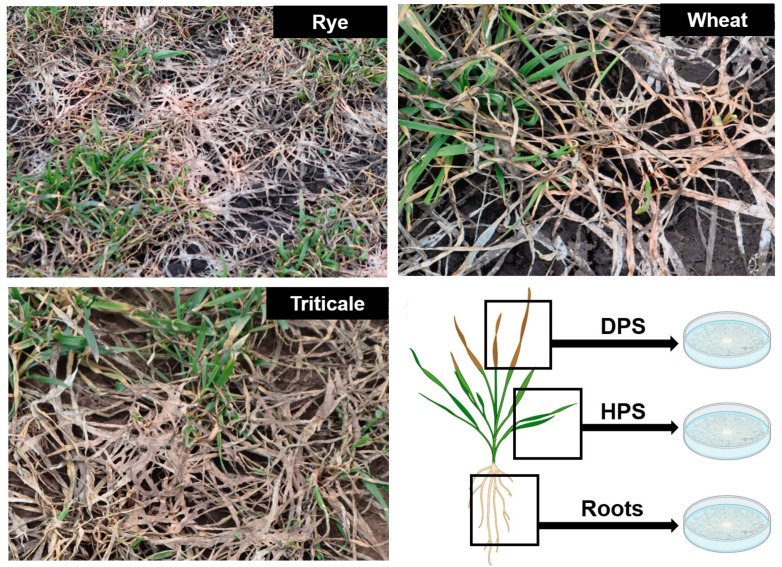
Winter cereal crops (rye, wheat, and triticale) affected by snow mold and a scheme of sample collection. DPSs—dead parts of shoots; HPSs—healthy parts of shoots.

Plant samples for isolating *M. nivale* strains were collected from two agrocenoses located in the Tatarstan Republic: (1) Bolshiye Kaban in the Laishevo district (latitude 55.625164 N, longitude 49.351334 E) and (2) Nalasa in the Arsk district (latitude 56.113468 N, longitude 49.774500 E). The two agrocenoses, hereafter referred to as Arsk and Laishevo, exhibited distinct soil–climatic and agrotechnological conditions (see Table S1 for details). Samples were taken from three winter cereal crops: winter rye (*Secale cereale* L. cv. Ogonek), winter wheat (*Triticum aestivum* L. cv. Nadezhda), and winter triticale (*Triticosecale* Wittm. cv. Kornet) (Figure 1). In both agrocenoses, these crops were grown under uniform agronomic management (including fertilizer and herbicides) in competitive variety field trials. Plant samples were collected one week after snowmelt. For each plant, three types of samples were collected: (1) roots, (2) healthy parts of shoots (HPSs), and (3) dead parts of shoots (DPSs) (Figure 1). Within each sample type, two plants from each of four randomized plots were harvested.

### 2.2. Isolation of M. nivale Strains

Samples of roots, HPSs, and DPSs of winter cereal crops (rye, wheat, and triticale) were washed several times with distilled water to remove all soil, then held in 70% ethanol for 10 s, and then washed twice with sterile distilled water. Next, 1.0–1.5 mm fragments of the sterilized plant material were placed in Petri dishes containing potato sucrose agar (PSA) [49] with 200 μg/mL gentamicin. After 5–10 days of incubation at +4 °C in darkness, small fragments of mycelium resembling that of *M. nivale* were transferred to fresh PSA medium with gentamicin. Following 3–7 days of cultivation at 20 °C, actively growing hyphae from the edge of the colony were transferred to fresh PSA medium with gentamicin; this procedure was repeated at least twice.

### 2.3. DNA Extraction, DNA Library Preparation, and Sequencing

Total DNA was extracted from the samples using a DNeasy PowerBiofilm Kit (Qiagen, Hilden, Germany) and Fast Prep × 24 homogenizer (MP Biomedicals, Solon, OH, USA) according to the protocol provided by the manufacturer. The quantity of extracted DNA was evaluated using a NanoDrop 2000 spectrophotometer (Thermo Fisher Scientific, Waltham, MA, USA). Four genome loci were sequenced in each strain: the internal transcribed spacer 2 (ITS2), a fragment of the elongation factor 1α gene (*EF-1α*), a fragment of the β-tubulin gene (*β-Tub*), and a fragment of the RNA polymerase II gene (*RpoL*). The ITS2 region of the fungal ribosomal RNA (rRNA) locus was amplified using ITS3_KYO2 (5′–GAT GAA GAA CGY AGY RAA–3′) and ITS4 (5′–TCC TCC GCT TAT TGA TAT GC–3′) primers [50].

Primers for the amplification of *M. nivale EF-1α* (MicEF_F1: 5′–GGT GAG TTC GAG GCT GGT ATC–3′, MicEF_R1: 5′–CAG GGG GCG TTG GTG G–3′), *β-Tub* (MicTub_F1: 5′–ACG CTC CTC ATC TCC AAG ATC–3′, MicTub_R1: 5′–GAA ACG CAG GCA GGT GGT–3′), and *RpoL* (MicRpol_F2: 5′–GCT TCC TGA TGT GCT CCT TG–3′, MicRpol_R2: 5′–GAA CCT TTC TCT TAT GTG CTC GA–3′) were designed in the present study. The primers were designed using Vector-NTI Version 9 software (Invitrogen, Carlsbad, CA, USA) based on the sequences available in NCBI for target loci in *M. nivale* and its closely related species *M. majus* to ensure amplification of a fragment of less than 450 bp that includes single-nucleotide polymorphisms that (1) enable differentiation between *M. nivale* and *M. majus*, and (2) reflect well the variability in target loci at the intraspecific level.

All DNA libraries were prepared according to the Illumina protocol (Illumina protocol, part no. 15044223, Rev. B). Amplification of the target fragments was carried out using Q5 high-fidelity DNA polymerase (NEB, Ipswich, MA, USA). The PCR products were purified with AMPure XP magnetic beads (Beckman Coulter, Indianapolis, IN, USA). The indexing of libraries was performed using the Nextera XT Index Kit v2 (Illumina, San Diego, CA, USA), followed by purification of the indexed libraries with AMPure XP magnetic beads. The libraries were then pooled and sequenced on the MiSeq platform using the MiSeq Reagent Kit v3 (600-cycles) (Illumina).

### 2.4. Bioinformatic Procedures

The obtained reads were processed using FastQC [51], MultiQC [52], and Cutadapt v.3.5 [53] for quality control and primer sequence removal. The DADA2 [54] pipeline was used for quality trimming, dereplication, filtering for chimeras, and generation of amplicon sequence variants (ASVs). The annotation of ASVs was performed using the National Center for Biotechnology Information (NCBI) database. The sequences of ITS2, as well as fragments of *EF-1α*, *β-Tub*, and *RpoL* genes analyzed in this study are deposited in the NCBI database (accession numbers PQ516916-PQ516917, PQ538502-PQ538508).

### 2.5. Construction of a Multigene Phylogenetic Tree

Along with the strains characterized in our study, previously characterized *M. nivale* and *M. majus* (outgroup) strains, for which all four target genome loci (ITS2, *EF-1α*, *β-Tub*, and *RpoL*) had been sequenced, were included in the multigene phylogenetic analysis. A list of these strains, along with their sequence accession numbers, is presented in Table S2. The ITS2, *EF-1α*, *β-Tub*, and *RpoL* nucleotide sequences for these strains were retrieved from the NCBI nucleotide database. For constructing the multigene phylogenetic tree, 263 bp fragments of ITS2, 226 bp fragments of *EF-1α*, 243 bp fragments of *β-Tub*, and 340 bp fragments of *RpoL* from *M. nivale* and *M. majus* were used. The phylogenetic tree was reconstructed using the IQTREE version 1.6.12 [55,56] with the maximum likelihood method with an ultrafast bootstrap (1000 replicates), rooted at the midpoint, and visualized using MEGA11. Individual models for determining the distance were automatically selected for each locus: K2P+I for ITS2, TNe+I for *EF-1α*, K2P for *RpoL*, and HKY+F+I for *β-Tub*.

### 2.6. Growth Rate Measurement

To assess the growth rates of the isolated strains, 7 mm diameter mycelial plugs, cut from the periphery of 10-day-old cultures, were placed in the center of Petri dishes containing PSA medium with 200 μg/mL gentamicin. The fungal cultures were cultured in darkness at 20 °C for 2 weeks. Every day, two perpendicular radii of each fungal colony were measured, and the average of these two radii was used to estimate the fungal growth rate. The growth rate of each strain was calculated by determining the slope of the linear regression obtained from plotting the change in colony radius over time. Each strain was analyzed in three biological replicates.

### 2.7. Virulence Assay

The virulence of isolated *M. nivale* strains toward various winter cereal crops (winter rye cv. Ogonek, winter wheat cv. Nadezhda, and winter triticale cv. Kornet) was evaluated using two methods: whole-plant assay (WPA) and detached-leaf assay (DLA).

WPA tests were conducted as follows. For sterilization, the seeds were initially soaked in room temperature water for 2 h, followed by treatment with a 5% of AgNO_3_ solution for 15 min. After the AgNO_3_ solution was removed, the seeds were washed five times with sterile 1% NaCl solution, with each wash lasting 1 min. Finally, the seeds were rinsed with sterile water. The seeds were germinated for 36 h at 28 °C in darkness. Seedlings were transferred to individual sterile 50 mL glass tubes with 10 mL of ¼ diluted Murashige and Skoog medium without organic carbon. Twelve hours later, the seedlings were infected with the isolated strains by placing an 8 mm mycelial plug (cut from the periphery of 7–10-day-old cultures grown on PSA) into the tube in contact with the seedling. For the control plants, 8 mm plugs of sterile PSA were used instead of the mycelial plugs. Both control and infected plants were grown at 20 °C with a 16 h light/8 h dark cycle photoperiod for 20 days. At least 20 biological replicates were analyzed for each strain on each crop. Twenty days after infection, the experimental (control and infected) plants were harvested, and the dry weight of roots was measured. The virulence of each strain was expressed as the reduced root dry weight (RRDW, %) of infected plants compared to control plants; a greater reduction in root dry weight indicated higher strain virulence.

For conducting the DLA tests, the plants were grown in soil in a glasshouse for 10 days at 20 °C with a 16 h light/8 h dark cycle photoperiod and 60% relative humidity. The leaves were then cut and 3 cm leaf fragments were placed in plastic containers on microscope slides; half of the fragments were positioned with the upper (adaxial) side up, and the other half with the lower (abaxial) side up. The leaves were pressed on both sides with two layers of absorbent cotton soaked in 0.004% aqueous benzimidazole solution. To prevent drying, the leaves were sprayed with sterile water. Infection of leaf segments with the analyzed strains was performed by placing an 8 mm mycelial plug (cut from the periphery of 7–10-day-old cultures grown on PSA) in the center of each leaf segment. Sterile PSA plugs were placed on the control leaf segments. The containers were covered with glass plates and incubated in a Binder chamber 720 MK (E5) (Germany) at 20 °C with a 16 h light/8 h dark photoperiod and 60% relative humidity. Disease lesions, measured in mm, were assessed individually in 30 biological replicates at 2-day intervals (4, 6, 8, and 10 days post-infection). The virulence of each strain was measured by calculating the area under the disease progress curve (AUDPC) according to the following formula:

AUDPC = ½ (x_1_ + x_2_)(t_2_ − t_1_) + … + (x_n−1_ + x_n_)(t_n_ − t_n−1_)

where AUDPC—the area under disease progress curve;

n—number of observations;

x_1_, x_2_, x_n_—the percentage of visible infected area at the time of the first, second, and further registration, %;

t_2_ − t_1_—the time between the second and first registration in days;

t_n_ − t_n-1_—the time between last and next-to-last registration in days.

### 2.8. Statistics

The results were analyzed using XLSTAT Statistical Analysis Software (version 2020.4.1.1018). The significance of differences between samples was assessed using the Mann–Whitney test (*p* < 0.05) or Mann–Whitney test with Bonferroni correction for multiple comparisons (FDR < 0.05). Spearman’s correlation coefficients and their statistical significance (*p*-value < 0.05) were calculated to examine the relationships between different phenotypes. Violin plots were generated using OriginPro 2021. To assess the variability in/similarity of virulence profiles among the analyzed strains, a K-means clustering analysis was performed. The number of clusters was chosen based on the elbow method. Relationships between genetic traits and the origin of all analyzed strains, as well as strains within each separate population, were assessed using the χ^2^ test (*p*-value < 0.05).

## 3. Results

### 3.1. Genetic Variability of M. nivale

We isolated 136 *M. nivale* strains from the roots, healthy parts of shoots (HPSs), and dead parts of shoots (DPSs) of three winter cereal crops (rye, wheat, and triticale) grown in two agrocenoses (Arsk and Laishevo) (Table 1).

**Figure 2:**
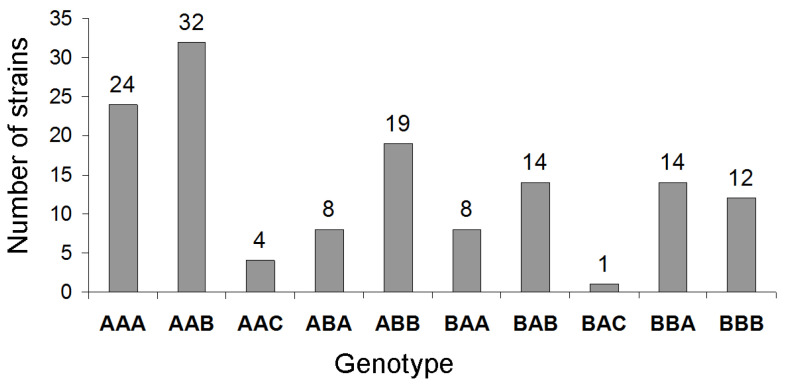
Number of *M. nivale* strains within ten revealed *M. nivale* genotypes. Genotypes are designated in three letters, where the first letter reflects the ITS2 variant (A/B), the second letter reflects the *EF-1α* variant (A/B), and the third letter reflects the *β-Tub* variant (A/B/C).

To verify that the isolated strains belonged to the *M. nivale* species and to assess the genetic diversity of the strains, four genome loci, namely, ITS2, *EF-1α*, *β-Tub*, and *RpoL*, were sequenced in each strain. Sequence analysis showed that all 136 strains belonged to the *M. nivale* species. In different strains, we identified two variants of the ITS2 sequence, referred to as ITS2_A (87 strains) and ITS2_B (49 strains), two variants of the *EF-1α* sequence, referred to as *EF-1α*_A (83 strains) and *EF-1α*_B (53 strains), and three variants of the *β-Tub* sequence, referred to as *β-Tub*_A (54 strains), *β-Tub*_B (77 strains), and *β-Tub*_C (5 strains). All 136 strains had identical *RpoL* sequences. The variants of sequences of different loci in the assayed strains were revealed in ten distinct combinations, which were further considered as ten *M. nivale* genotypes (Figure 2).

**Figure 3:**
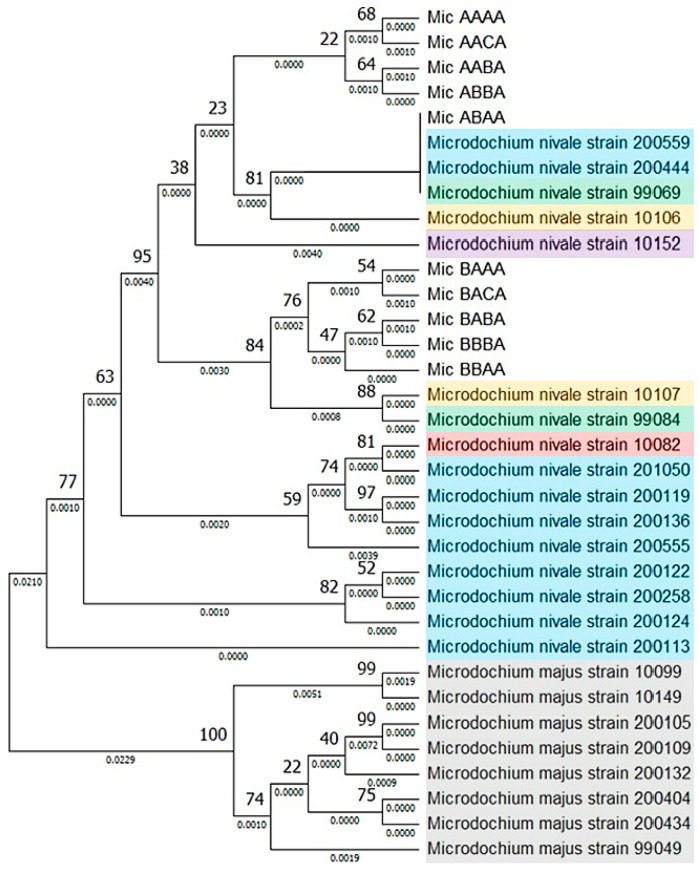
A multigene phylogenetic tree for the strains of *M. nivale* and the closely related species *M. majus* (outgroup) built based on the sequences of the internal transcribed spacer 2 (ITS2), a fragment of the elongation factor 1α gene (*EF-1α*), a fragment of the β-tubulin gene (*β-Tub*), and a fragment of the RNA polymerase II gene (*RpoL*). The phylogenetic tree was reconstructed using the maximum likelihood method with an ultrafast bootstrap (1000 replicates), rooted at the midpoint, and visualized using MEGA11. Bootstrap values are indicated above the nodes, and branch lengths are indicated below the branches. Ten genotypes comprising 136 strains isolated and characterized in this study are indicated as Mic with four capital letters corresponding to the variants of the four sequenced genome loci: the first letter reflects the ITS2 variant (A/B), the second letter reflects the *EF-1α* variant (A/B), the third letter reflects the *β-Tub* variant (A/B/C), and the fourth letter reflects the only *RpoL* variant (A). Blue, red, yellow, purple, and green backgrounds mark previously assayed *M. nivale* strains in which all four target genome loci were previously sequenced (the list of these strains with sequence accession numbers is presented in Table S2); these strains were isolated in Norway (blue), Great Britain (red), Italy (yellow), France (purple), and Canada (green). Strains of the closely related species *M. majus* (outgroup) are marked with a gray background.

To assess the degree of the genetic similarity/variability among the ten *M. nivale* genotypes revealed as well as between these genotypes and *M. nivale* strains from other countries for which all four analyzed DNA fragments were sequenced (Table S2), a multigene phylogenetic tree was built. The ten *M. nivale* genotypes revealed were located within three subclades (Figure 3). Four genotypes (designated AAAA, AACA, AABA, ABBA according to the variants of the four sequenced loci) formed the first subclade that did not contain strains from other countries. One genotype (ABAA), represented by eight strains, formed the second subclade together with three strains (200559 from Norway, 200444 from Norway, 99069 from Canada) that are identical to the ABAA-genotype sequences across all four loci and one closely related strain (10106 from Italy). The low bootstrap value suggests that the strains in the first and second subclades are likely to be closely related genetically. Five genotypes (BAAA, BACA, BABA, BBBA, BBAA) formed the third subclade, which was located close to the subclade formed by strains 10107 and 99084 from Italy and Canada, respectively (Figure 3).

### 3.2. Phenotypic Variability of the M. nivale Strains

**Figure 4:**
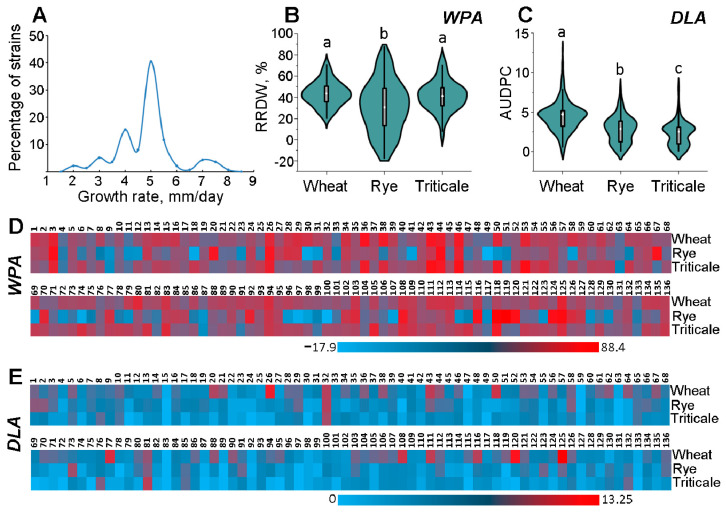
Phenotypic characteristics of the 136 *M. nivale* strains. (**A**) Frequency distribution of growth rates in *M. nivale* assessed by grouping strains into 15 groups differing in growth rates by 0.5 mm/day. Growth rates were measured in five biological replicates for each strain. (**B**,**C**) Distribution of virulence of the 136 *M. nivale* strains toward winter crops (wheat, rye, and triticale) assessed by two methods: whole-plant assay (WPA) (**B**) and detached-leaf assay (DLA) (**C**). In the WPA tests, virulence was expressed as reduced root dry weight (RRDW, %) of infected plants compared to control non-infected plants; negative values indicate that some strains caused a positive effect on rye root biomass. The virulence of each strain was analyzed in twenty biological replicates. In DLA-tests, virulence was expressed as the area under the disease progress curve (AUDPC) and analyzed in 30 biological replicates. Different letters on the bars in (**B**,**C**) panels indicate significant differences (Mann–Whitney test with Bonferroni correction for multiple comparisons (FDR < 0.05)). (**D**,**E**) Heat maps reflecting virulence profile (virulence to each of the three crops) of each particular strain assessed by WPA (**D**) and DLA (**E**) tests.

To estimate the phenotypic variability of the 136 analyzed *M. nivale* strains, the growth rates in vitro were determined, and virulence toward three winter cereal crops (rye, wheat, and triticale) was assessed using two methods: whole-plant assay (WPA) and detached-leaf assay (DLA). The growth rates of the strains varied from 1.7 to 7.6 mm per day, with 40% of the strains showing a growth rate of 4.5–5.0 mm per day (Figure 4A).

**Figure 5:**
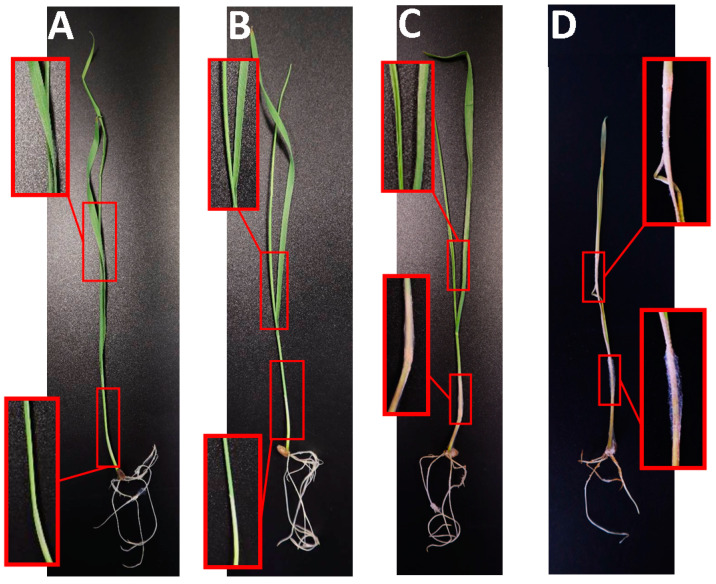
Winter wheat plants non-infected (**A**) or infected with the *M. nivale* strains (**B**–**D**) exhibiting different virulence levels: avirulent (**B**), moderately virulent (**C**), and highly virulent (**D**). The photos were taken 20 days post infection.

In WPA tests, the *M. nivale* strains displayed a strong difference in virulence (Figure 5), causing various levels of root growth retardation as expressed in reduced root dry weight (RRDW) of the infected plants compared to the control plants. In wheat, the RRDW ranged from 20 to 73%, in rye, from −18 to 88%, and in triticale, from 3 to 77% (Figure 4B). The negative RRDW values for rye indicated that some of the strains enhanced root development (up to 18% compared to the non-infected control), and rye was the only crop for which the positive effect of particular *M. nivale* strains on root growth was revealed. In WPA tests, rye suffered from *M. nivale* significantly less than wheat and triticale, and the highest range of values of *M. nivale* virulence was also revealed in rye (Figure 4B). In DLA tests, strains also displayed differences in virulence (Figure 4C). The crop least affected by *M. nivale* in the DLA tests was triticale, whereas wheat was the most affected crop (Figure 4C).

Different *M. nivale* strains were characterized by both similar and differing virulence toward different crops. For example, in WPA tests, strains Nos. 3, 26, 44, 46, and 125 had high virulence, whereas strains Nos. 9, 87, and 117 had low virulence toward all three crops; strains Nos. 20, 67, 70, 92, and 119 had high virulence toward rye and low virulence toward wheat and triticale, whereas strains Nos. 36, 38, 50, 58, 69, and 79 had high virulence toward wheat and triticale and low virulence toward rye (Figure 4D). Both similar and differing virulence of different strains toward different crops were also revealed in DLA tests (Figure 4E).

**Figure 6:**
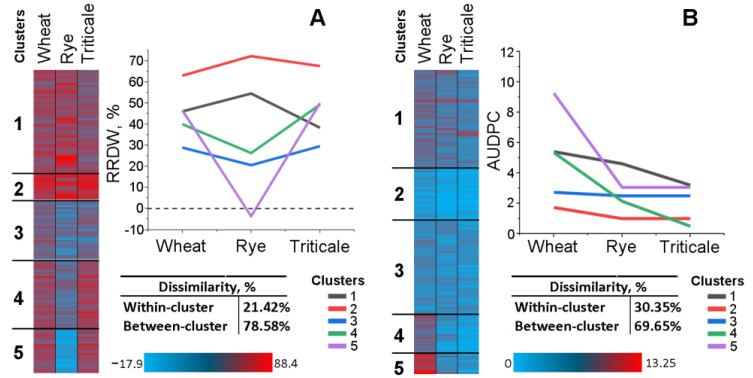
K-means clustering of 136 *M. nivale* strains based on the parameters of their virulence toward winter crops (wheat, rye, and triticale) assessed by whole-plant assay (WPA) (**A**) and detached-leaf assay (DLA) (**B**). The proximity between objects was measured based on Euclidian distance.

To verify whether the analyzed strains were split into relatively dissimilar groups in terms of their virulence profiles (virulence toward different crops), k-means clustering between the strains’ virulence parameters assessed by WPA and DLA was performed. According to WPA, the analyzed 136 strains were divided into five clusters based on the parameters of their virulence toward different winter crops (Figure 6).

The first (the most represented) cluster included 47 moderately virulent strains that caused root growth retardation expressed with 40–50% reduced root dry weight (RRDW) compared to the control in all three crops. The second and third clusters included 12 highly virulent and 27 low virulent strains, respectively, which caused reductions in root dry weight by more than 60% and less than 30% (on average for the cluster), respectively, in all three crops. Strains of the fourth and fifth clusters displayed differential virulence toward different crops being more virulent toward wheat and triticale than toward rye. Of particular interest were 19 strains from the fifth cluster that displayed moderate virulence to wheat and triticale and avirulence toward rye, with most of the strains increasing rye root dry weight (presenting negative RRDW values) up to 18% (Figure 6).

According to DLA, the analyzed 136 strains were also divided into five clusters (Figure 6). The first and the third clusters included 43 and 42 strains with moderate and low virulence, respectively, toward all three crops, whereas the second cluster included 25 almost avirulent strains. Strains of the fourth and fifth clusters displayed differential virulence toward different crops. Seventeen strains of the fourth cluster displayed moderate and low virulence to wheat and rye, respectively, and were almost avirulent toward triticale, whereas nine strains of the fifth cluster displayed low virulence toward rye and triticale and simultaneously were highly virulent toward wheat (Figure 6).

### 3.3. Analysis of Potential Relationships Between Various Phenotypic Traits of M. nivale Strains

Then, we assessed whether correlations exist between different phenotypic traits of strains or between phenotypic traits and origin of strains. We revealed that a significant weak correlation exists between the virulence of strains assessed using WPA and DLA, and this weak correlation was revealed with regard to rye and wheat, but not triticale plants (Table 2).

Since strains differed in their growth rate in vitro, we analyzed whether the growth rate had a correlation with virulence assessed on three crops by two methods. No significant correlation between growth rate and virulence was observed except that growth rate had a tenuous (but significant) correlation with virulence assessed by WPA on triticale (Table 2). Given the differential virulence of strains on different crops (see above), for each strain, we selected one maximal virulence value (determined on the crop on which virulence was most pronounced for the strain) (differentially for WPA and DLA), and assessed the correlation between the growth rate and maximal virulence value (irrespective of the crop on which maximal virulence level was manifested). No significant correlation was found between maximal virulence and growth rate with Spearman’s correlation coefficients of −0.103 and −0.067 for WPA and DLA, respectively.

The relationships between the virulence of strains on different crops were revealed. In WPA, the virulence on wheat had significant weak and moderate positive correlations with virulence on rye and triticale, respectively, whereas in DLA, a significant weak to moderate positive correlation was revealed in virulence on all three crops (Table 2). The analysis did not reveal any relationships between virulence (toward each of the three crops assessed by either WPA or DLA) and the origin of strains: original host (rye, wheat, or triticale) or plant part from which a strain was isolated (roots, DPSs, or HPSs) (Figures S1 and S2).

### 3.4. Analysis of Potential Relationships Between Various Phenotypic and Genetic Traits of M. nivale Strains

We then analyzed whether genetic traits of *M. nivale* strains were associated with phenotypic traits (virulence toward different crops and growth rate) of the strains. As genetic traits, we considered which variant of each of the three analyzed sequences (ITS2 (A or B), *EF-1*α (A or B), or *β-Tub* (A, B, or C)) was typical of a strain and to which of the nine genotypes a strain belonged (the genotypes are described in Figure 2). One of the ten identified genotypes (BAC) was not considered because it included only one strain.

No relationships between virulence parameters (assessed differentially for all three crops by two methods: WPA and DLA) and genetic traits were revealed, except that strains with the “A” variant of *EF-1α* were less virulent toward wheat leaves in DLA tests than strains with the “B” variant of *EF-1α* (Figures S3 and S4). No relationships between genetic traits and growth rate of the strains were observed (Figure S5).

No relationships between genetic traits and the origin of strains (original host crop (rye, wheat, or triticale) or plant part from which a strain was isolated (roots, DPSs, or HPSs)) were revealed, except that strains with different variants of the β-tubulin gene sequence were isolated from rye and triticale plants at varying frequencies: strains with the “A” variant of the β-tubulin gene were isolated more frequently from triticale and less frequently from rye than expected, whereas strains with the “B” variant of the β-tubulin gene were isolated more frequently from rye and less frequently from triticale than expected (χ^2^-test, *p*-value < 0.05) (Supplementary File S1).

### 3.5. Comparison of Two M. nivale Populations in Terms of Phenotypic and Genetic Traits of Strains

We also analyzed whether *M. nivale* strains from two different populations differed in terms of genetic and/or phenotypic traits. In Laishevo, strains with the “B” variant of *β-Tub* were more prevalent than those with the “A” variant, whereas in Arsk, strains with the “A” variant of *β-Tub* were more common than those with the “B” variant. Consequently, there was a higher representation of “B” variant strains in Laishevo compared to Arsk, and a higher representation of “A” variant strains in Arsk compared to Laishevo (χ^2^, *p*-value < 0.05) (Supplementary File S2). No other differences in genetic traits were revealed between strains from different populations (Supplementary File S2).

In terms of the phenotypic traits, strains from Laishevo grew on average 20% faster than strains from Arsk (Figure S6). Here, it was strains with the “A” variant of *EF-1α* and the “B” variant of *β-Tub* from Laishevo that displayed faster growth rates compared to the strains with the corresponding variants of *EF-1α* and *β-Tub* from Arsk (Figure S7). At the genotype level, the AAB and BAB genotypes from Laishevo displayed faster growth rates compared to the corresponding genotypes from Arsk (Figure S7).

The two populations showed no significant difference in the distribution of strains with varying levels of virulence, regardless of the crop (rye, wheat, or triticale) used in the virulence assays or the type of assay employed (WPA or DLA) (Figure S8). Only a few relationships between virulence (toward rye, wheat, triticale assessed by WPA and DLA) and genetic traits (sequence variants of ITS2, *EF-1α*, *β-Tub*, or attribution to a particular genotype) were revealed for strains within each separate population: (1) strains from Laishevo with the BBA genotype exhibited higher virulence toward wheat in DLA tests compared to strains from Laishevo with the AAB genotype; (2) strains with the “B” variant of ITS2 from Arsk were more virulent toward triticale in WPA tests than strains with the “A” variant of ITS2 from Arsk; (3) strains with the “B” variant of *EF-1α* from Laishevo were more virulent toward wheat in DLA tests than strains with the “A” variant of *EF-1α* from Laishevo (Figures S9 and S10).

## 4. Discussion

In the present study, we assessed the intrapopulation and interpopulation genetic and phenotypic diversity of the phytopathogenic fungal species *M. nivale*. A total of 136 *M. nivale* strains were isolated from winter cereal crops (rye, wheat, and triticale) grown in two agrocenoses: 60 and 76 strains were collected from the Arsk and Laishevo agrocenoses, respectively. The strains were classified into 10 genotypes based on sequences of four phylogenetic markers: ITS2; *EF-1α*, *β-Tub*, *RpoL*. Each genotype represented 1–32 strains.

Most of the analyzed *M. nivale* strains (128 out of 136), representing nine of the ten *M. nivale* genotypes identified, formed two major subclades that were rather distant from the subclades formed by previously investigated strains from other countries, indicating that most of the isolated strains are specific to the studied area. Eight strains belonging to one of the identified genotypes were found to be closely related to strains from Norway and Canada, sharing identical sequences at all four analyzed genome loci. This suggests that strains of this genotype may have a global distribution, with the potential for inter-regional dispersal.

The tested strains demonstrated variability in their virulence, evaluated through both WPA—by measuring the reduction in root weight of infected plants compared to control plants, which enables more precise differentiation of strains based on their effect on the host plant than visual symptom assessment [48]—and DLA, as well as in their in vitro growth rates. We presumed that higher growth rates can be associated with higher virulence; however, no relationships were found between these two parameters, regardless of the crop used in the virulence assays (rye, wheat, or triticale) or the type of assay employed (DLA or WPA). This disconnect between in vitro growth rates and virulence has also been observed in other fungal phytopathogens [57,58,59,60,61], indicating that high growth rates do not necessarily contribute to high virulence.

The assessment of virulence using two distinct methods (DLA and WPA) resulted in different rankings of strains based on this trait: no significant correlations, or only weak correlations, were observed between the virulence of strains evaluated using different methods regardless of the crop employed. Some studies have reported discrepancies in the results of virulence assessments conducted on whole plants and detached leaves [62,63,64,65]. These discrepancies have been attributed to the differing reactions of whole plants and detached leaves to pathogen inoculation. However, some studies have demonstrated that the virulence of phytopathogenic fungal strains tends to be expressed similarly, regardless of whether whole plants or detached leaves are used for evaluation [60,61]. Thus, the presence or absence of correlations in this context likely depends on the specific pathogen–host combination. In the case of *M. nivale*, previous research has demonstrated that the expression of virulence can be influenced by the method of plant inoculation [43], which aligns with our observations.

The studied strains exhibited varying levels of virulence, with some displaying similar virulence across all three crops (either consistently high or consistently low), while others showed significant differences in virulence depending on the crop species. The majority of strains demonstrated similar levels of virulence across different crops, suggesting a weak expression of host preference in the studied *M. nivale* strains. This notion is supported by the observed correlations between strain virulence toward different crops. Additionally, no relationships were found between the initial host from which a strain was isolated and its virulence towards that host compared to other crops. A similar lack of increased virulence toward the initial host has been previously reported for *Sclerotinia sclerotiorum*: the virulence of *S. sclerotiorum* strains isolated from melon was not higher toward melon compared to the virulence of strains isolated from other crops [66].

On the one hand, our results appear to contradict previous studies indicating host preference in *M. nivale* strains; however, these earlier findings focused on host preference among different grass species or between grasses and cereals but not among different cereal crops [46,47]. It is also known that *M. nivale* exhibits relatively higher virulence toward rye and perennial grasses, whereas its close relative *M. majus* exhibits relatively higher virulence toward wheat, oat, barley, and triticale [42,67,68,69,70]. Nevertheless, it remains unclear whether different *M. nivale* strains exhibited differentiation in host preference among different cereal crops.

On the other hand, the elements of host preference were observed in our study for certain strains. In this context, a notable group of strains stood out, exhibiting moderate virulence toward wheat and triticale, while being completely avirulent toward rye, even stimulating the growth of rye’s root system. It would be interesting to explore whether these strains are unable to interact with rye, in contrast to wheat and triticale, or whether they interact with wheat and triticale in a more parasitic way and with rye in a more mutualistic way, thereby employing different interaction strategies with different crops. The latter hypothesis is supported by our recent study, which demonstrated that although snow mold damage is more pronounced in wheat and triticale than in rye under field conditions, rye is more intensively colonized by *M. nivale* [71]. This finding is consistent with the fact that rye is regarded as the most resistant crop to snow mold [21,72]. Further comparative research on the “more parasitic” and “more mutualistic” aspects of *M. nivale* interactions with plants will provide a foundation for improved disease management, and our study contributes to these investigations.

Associations between the studied genetic and phenotypic traits were found among the strains only in individual cases: (1) strains with the “A” variant of *EF-1α* were less virulent toward wheat leaves in DLA tests compared to strains with the “B” variant of *EF-1α*, and (2) strains with the “A” variant of the β-tubulin gene were isolated more frequently from triticale and less frequently from rye, whereas the opposite was true for strains with the “B” variant. The limited association between the studied genetic and phenotypic traits is consistent with the fact that relationships between phylogenetic markers (to which the assayed loci belong) and phenotypic features are typically not detected [12,14,60,73]. These genetic markers allow for the assessment of genetic diversity and the degree of relatedness among different strains. However, within a specific phylogenetic group, strains exhibit significant differences in their phenotypic characteristics [12,60,74], a finding that was also evident in our study. Furthermore, phylogenetic markers sometimes enable differentiation among phytopathogen populations [12,15,47,75], a result that was also achieved in our study. In order to comprehend the genetic basis of the strains’ differential behavior (specifically among those within the same phylogenetic group), it is necessary to identify genetic markers of phenotypic traits, and the recently deciphered genome sequence of *M. nivale* represents a significant contribution to solving this task [76]. In turn, the strains tested in our study, with their high genetic and phenotypic variability, offer a robust platform for identifying markers that distinguish specific strains with contrasting traits at the whole-genome level, which can subsequently be validated in a large, diverse sample of strains from a particular population. The identification of genetic markers of phenotypic traits will lay the groundwork for the development of enhanced diagnostic approaches, facilitating objective evaluations of epiphytotic situations.

The genetic and phenotypic profiles of the two studied *M. nivale* populations were largely similar, despite high levels of diversity within each population. This aligns with previous studies showing that intrapopulation diversity contributes more to intraspecific diversity of phytopathogenoc fungi than interpopulation diversity [1,4,5,6,7,8,9,10,11,12,13]. At the same time, populations of several species of phytopathogenic fungi, including *M. nivale*, have been shown to manifest distinctive “geographic” features [1,2,3,4,47]. We also found genetic differences between the studied populations: strains with the “A” variant of β-tubulin were more prevalent in the Arsk population, whereas strains with the “B” variant of β-tubulin were more common in the Laishevo population. Uncovering the biological significance of these between-population differences is one of the tasks for our further research.

## 5. Conclusions

*Microdochium nivale* strains colonizing winter cereal crops exhibit significant genetic and phenotypic diversity. Of the 136 assayed strains, most possess distinctive genetic features compared to previously characterized strains from other countries. The studied *M. nivale* populations include both highly virulent and lowly virulent strains, with the majority of the strains displaying similar virulence levels (either similarly high or similarly low) across different host crops. However, some strains demonstrate differential virulence toward different crops; of particular interest are those that cause disease in wheat and triticale while remaining avirulent toward rye, where they may even exhibit a plant-beneficial effect. Further research of these strains will improve our understanding of the criteria that distinguish “more parasitic” from “more mutualistic” behaviors of *M. nivale*.

No strong relationships were found between the studied genetic and phenotypic traits. Based on the initial host crop from which a strain was isolated, the strains did not differ in almost all examined parameters, except that strains with the “A” and “B” variants of the β-tubulin gene were more frequently isolated from triticale and rye, respectively. Despite the similarities between the two studied populations in most of the examined genetic and phenotypic traits, it was observed that different populations had varying ratios of strains with distinct β-tubulin sequence variants. Understanding the biological significance of this difference will be one of the objectives of our further research, alongside the search for genetic markers of phenotypic traits in the studied strains. Identifying such markers will facilitate the development of reliable diagnostics for predicting crop loss risk, both for cereals in general and for specific crops, thereby improving disease management. Furthermore, the most virulent strains we isolated will aid in identifying reliable sources of resistance within the winter cereal gene pool, which is currently the focus of our research and practical plant breeding [72].

## Figures and Tables

**Table 1:** Number of the *M. nivale* strains (136 in total) isolated from the roots, healthy parts of shoots (HPSs), and dead parts of shoots (DPSs) of three winter crops (rye, wheat, and triticale) grown in two agrocenoses (Arsk and Laishevo).

Agrocenosis	Arsk			Laishevo		
Plant part	Roots	HPSs	DPSs	Roots	HPSs	DPSs
Rye	6	4	20	10	12	12
Wheat	0	2	5	0	2	9
Triticale	0	11	12	3	10	18

**Table 2:** Spearman’s correlation between the virulence of the *M. nivale* strains on different winter cereal crops (wheat, rye, and triticale) (blue background), between the virulence of the *M. nivale* strains on a particular crop assessed by different methods (WPA, DLA) (red background), or between the growth rate and virulence (assessed on different crops by different methods) of the *M. nivale* strains (yellow background). Significant correlations (*p* < 0.05) are marked in bold and with asterisks. WPA—whole-plant assay; DLA—detached-leaf assay.

Variables	WPA Wheat	WPA Rye	WPA Triticale	DLA Wheat	DLA Rye	DLA Triticale
WPA_Wheat	**1**					
WPA_Rye	0.336 *	**1**				
WPA_Triticale	0.541 *	0.114	**1**			
DLA_Wheat	0.362 *	0.375 *	0.251 *	**1**		
DLA_Rye	0.246 *	0.266 *	0.079	0.496 *	**1**	
DLA_Triticale	0.155	0.129	−0.024	0.346 *	0.690 *	**1**
Growth rate	0.065	0.104	0.180 *	−0.065	−0.054	−0.042

## Data Availability

The described DNA sequences are available at the NCBI (accession numbers PQ516916-PQ516917, PQ538502-PQ538508).

## Supplementary Materials

The following supporting information can be downloaded at: https://www.mdpi.com/article/10.3390/jof10120841/s1, Figure S1: The virulence of *Microdochium nivale* strains isolated from different original host plants (rye, wheat, or triticale) toward each of the three crops; Figure S2: The virulence of *Microdochium nivale* strains isolated from different samples (roots, healthy parts of shoots (HPSs), and dead parts of shoots (DPSs)) of winter crops (rye, wheat, and triticale) toward each of the three crops; Figure S3: The virulence of *Microdochium nivale* strains with different sequence variants of the three loci: the internal transcribed spacer 2 (ITS2) (A or B), a fragment of the elongation factor 1α gene (*EF-1α*) (A or B), a fragment of the β-tubulin gene (*β-Tub*) (A, B, or C) toward three winter cereal crops (rye, wheat, and triticale); Figure S4: The virulence of *Microdochium nivale* strains belonging to nine different genotypes toward three winter cereal crops (rye, wheat, and triticale); Figure S5: The growth rates of *Microdochium nivale* strains belonging to nine different genotypes and strains with different sequence variants of the three loci: the internal transcribed spacer 2 (ITS2) (A or B), a fragment of the elongation factor 1α gene (*EF-1α*) (A or B), a fragment of the β-tubulin gene (*β-Tub*) (A, B, or C) toward three winter cereal crops (rye, wheat, and triticale); Figure S6: The growth rates (**A**) and the distribution of growth rates (**B**) of *Microdochium nivale* strains from two populations: Laishevo (red) and Arsk (green); Figure S7: The growth rates of *Microdochium nivale* strains from two populations: Laishevo (La, red) and Arsk (Ar, green); Figure S8: The virulence of *Microdochium nivale* strains from two populations: Arsk (Ar) and Laishevo (La) toward three winter cereal crops (wheat (W), rye (R), and triticale (T)); Figure S9: The virulence of *Microdochium nivale* strains from two populations: Arsk (Ar) and Laishevo (La) toward three winter cereal crops (rye, wheat, and triticale); Figure S10: The virulence of *Microdochium nivale* strains from two populations: Arsk (Ar) and Laishevo (La) toward three winter cereal crops (rye, wheat, and triticale); Table S1: Comparison of the two agrocenoses considered in the present study; Table S2: Strains of *Microdochium nivale* and *Microdochium majus*, with corresponding accession numbers for sequences of the internal transcribed spacer 2 (ITS2), a fragment of the elongation factor 1α gene (*EF-1α*), a fragment of the β-tubulin gene (*β-Tub*), and a fragment of the RNA polymerase II gene (*RpoL*) used in multigene phylogenetic analysis; Supplementary File S1. Evaluation of the relationships between genetic traits of *Microdochium nivale* strains and strain origin using the χ^2^ method; Supplementary File S2: The comparison of the two populations of *Microdochium nivale* from Arsk and Laishevo regarding the representation of strains with alternative variants of the three analyzed genomic loci: ITS2 (A or B) (list ITS), *EF-1α* (A or B) (list EF), and β-tubulin (A, B, or C) (list TUB) using the χ^2^ method.
