# Supplementary material for: Intra- and Interpopulation Diversity of the Phytopathogenic Fungi of the Microdochium nivale Species
Source: J Fungi (Basel). 2024 Dec 5;10(12):841. doi: 10.3390/jof10120841 (PMC11678714; doi:10.3390/jof10120841)
Supplement: Supplementary file 1 [file jof-10-00841-s001.zip › Supplementary figures S1-S10.pdf]

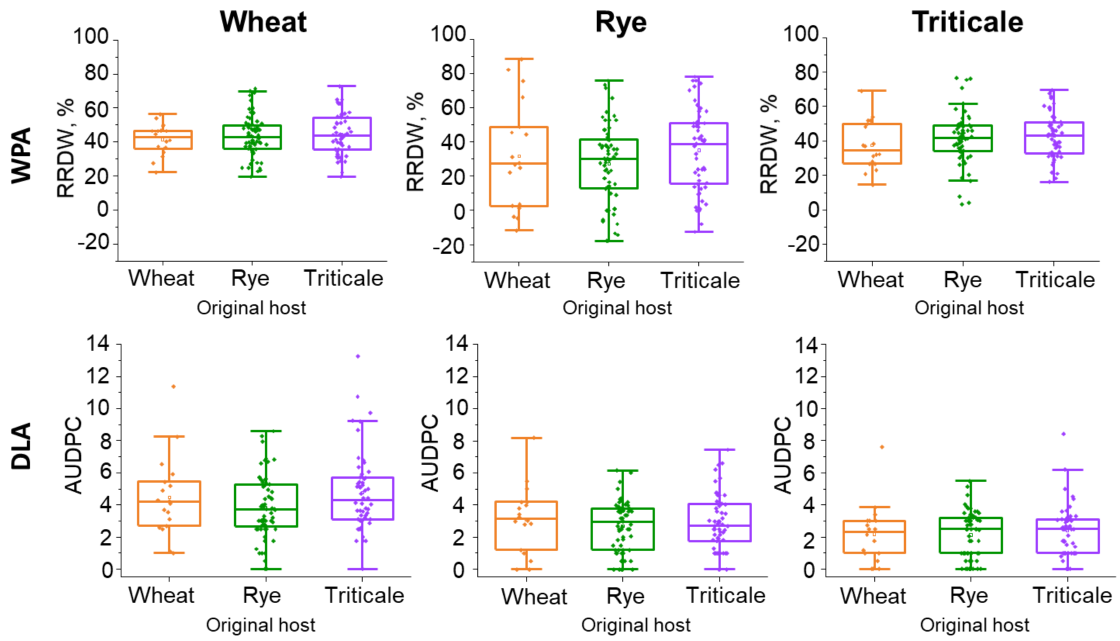

Figure S1. The virulence of *Microdochium nivale* strains isolated from different original host plants (rye, wheat, or triticale) toward each of the three crops. Virulence was assessed using the whole-plant assay (WPA) and expressed as reduced root dry weight (RRDW, %) of infected plants compared to control non-infected plants, as well as using detached leaf assay (DLA) and expressed as the area under the disease progress curve (AUDPC). No significant differences were revealed between the experimental groups (Mann-Whitney test with Bonferroni correction for multiple comparisons, FDR < 0.05).

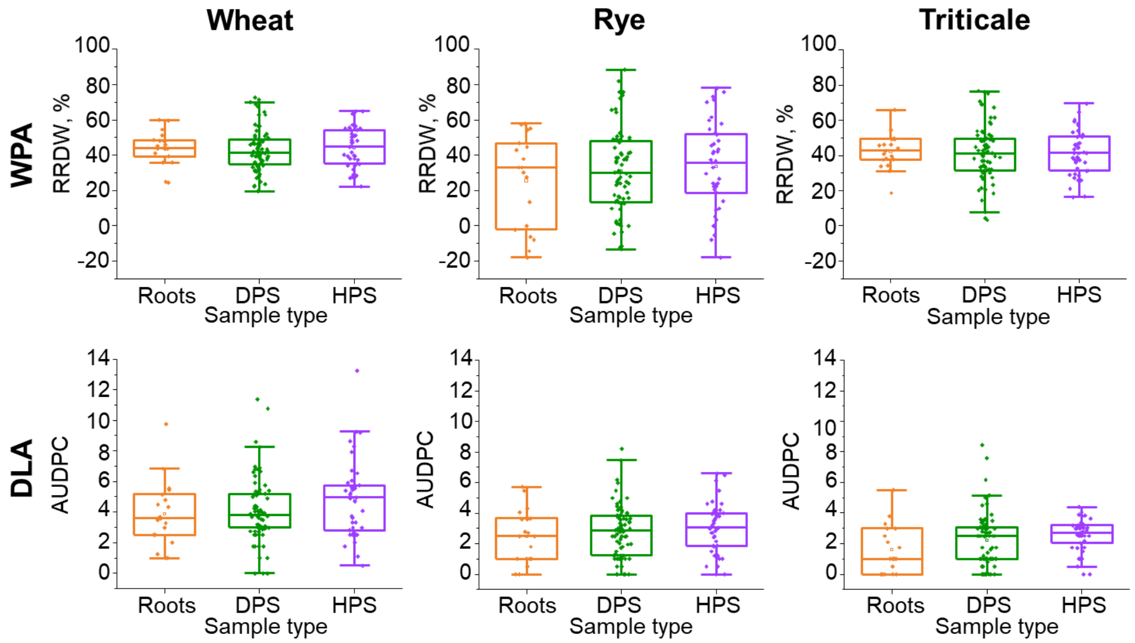

Figure S2. The virulence of *Microdochium nivale* strains isolated from different samples (roots, healthy parts of shoots (HPS), and dead parts of shoots (DPS)) of winter crops (rye, wheat, and triticale) toward each of the three crops. Virulence was assessed using the whole-plant assay (WPA) and expressed as reduced root dry weight (RRDW, %) of infected plants compared to control non-infected plants, as well as using detached leaf assay (DLA) and expressed as the area under the disease progress curve (AUDPC). No significant differences were revealed between the experimental groups (Mann-Whitney test with Bonferroni correction for multiple comparisons, FDR < 0.05).

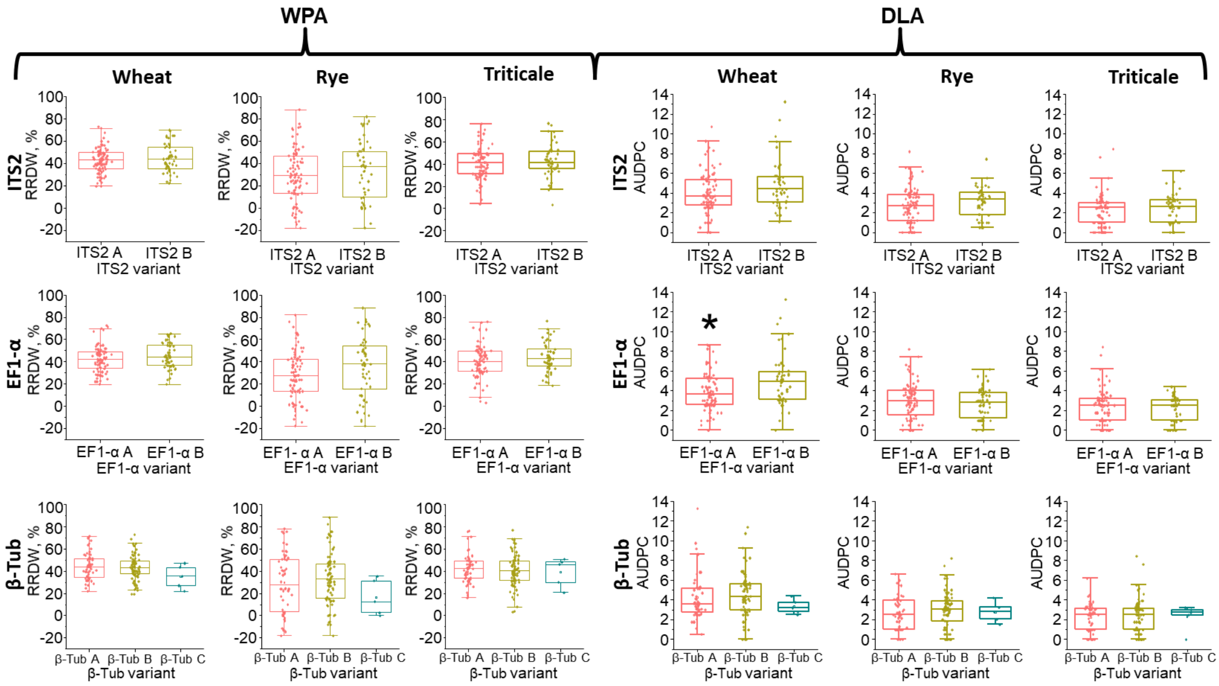

Figure S3. The virulence of *Microdochium nivale* strains with different sequence variants of the three loci: the internal transcribed spacer 2 (ITS2) (A or B), a fragment of the elongation factor 1α gene (EF-1α) (A or B), a fragment of the β-tubulin gene (β-Tub) (A, B, or C) toward three winter cereal crops (rye, wheat, and triticale). Virulence was assessed using the whole-plant assay (WPA) and expressed as reduced root dry weight (RRDW, %) of infected plants compared to control non-infected plants, as well as using detached leaf assay (DLA) and expressed as the area under the disease progress curve (AUDPC). An asterisk indicates a significant difference (Mann-Whitney test, p-value<0.05) between the virulence of strains with the "A" variant of EF-1α compared to the virulence of strains with the "B" variant of EF-1α toward wheat leaves in DLA tests.

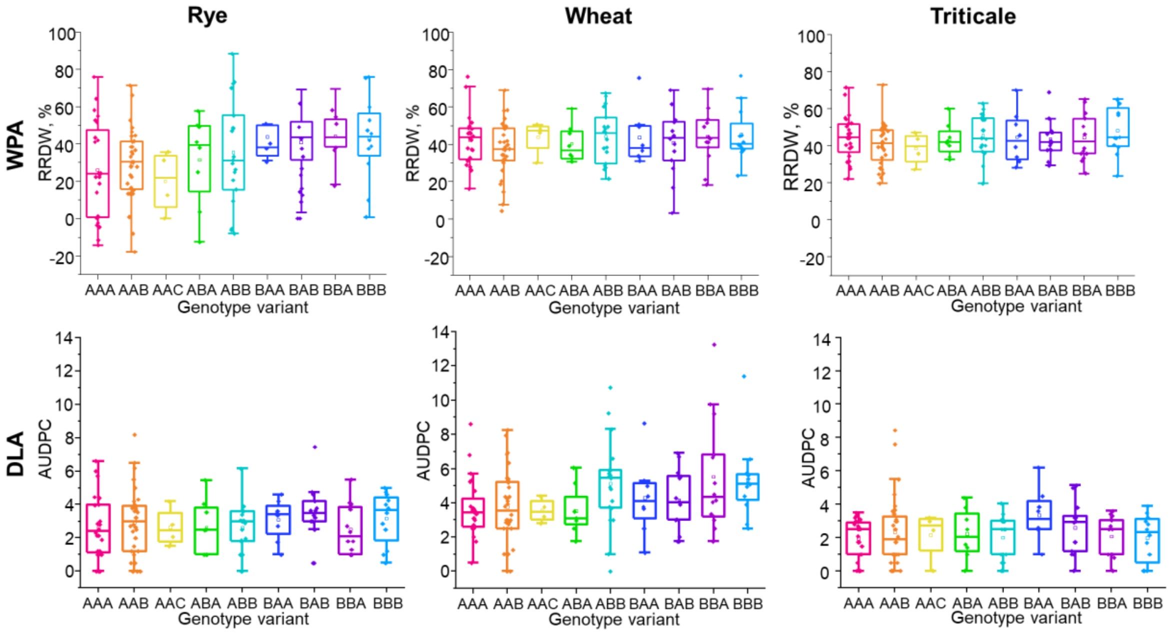

Figure S4. The virulence of *Microdochium nivale* strains belonging to nine different genotypes toward three winter cereal crops (rye, wheat, and triticale). Genotypes are designated in three letters, where the first letter reflects the ITS2 variant (A/B), the second letter reflects the EF-1α variant (A/B), and the third letter reflects the β-Tub variant (A/B/C). Virulence was assessed using the whole-plant assay (WPA) and expressed as reduced root dry weight (RRDW, %) of infected plants compared to control non-infected plants, as well as using detached leaf assay (DLA) and expressed as the area under the disease progress curve (AUDPC). No significant differences were revealed between the experimental groups (Mann-Whitney test with Bonferroni correction for multiple comparisons, FDR < 0.05).

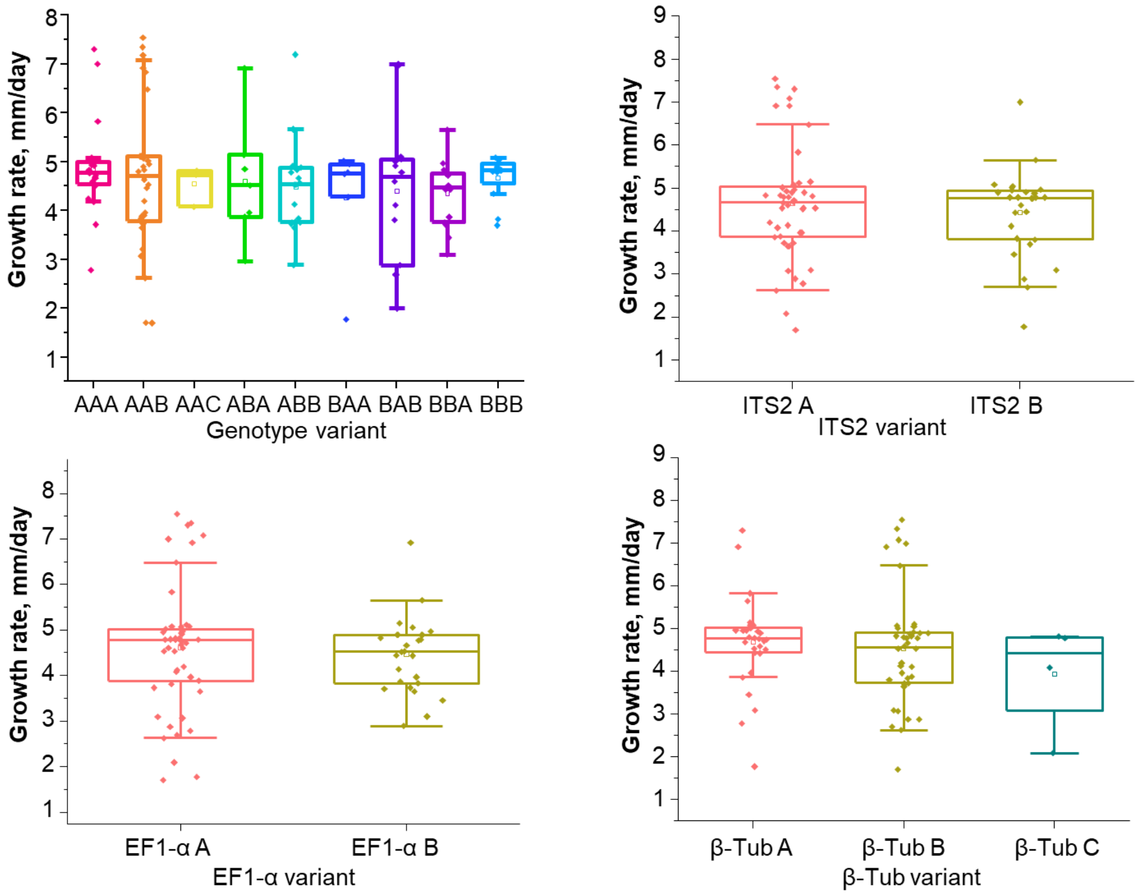

Figure S5. The growth rates of *Microdochium nivale* strains belonging to nine different genotypes and strains with different sequence variants of the three loci: the internal transcribed spacer 2 (ITS2) (A or B), a fragment of the elongation factor 1α gene (EF-1α) (A or B), a fragment of the β-tubulin gene (β-Tub) (A, B, or C) toward three winter cereal crops (rye, wheat, and triticale). Genotypes are designated in three letters, where the first letter reflects the ITS2 variant (A/B), the second letter reflects the EF-1α variant (A/B), and the third letter reflects the β-Tub variant (A/B/C). No significant differences were revealed between the experimental groups (Mann-Whitney test with Bonferroni correction for multiple comparisons, FDR < 0.05).

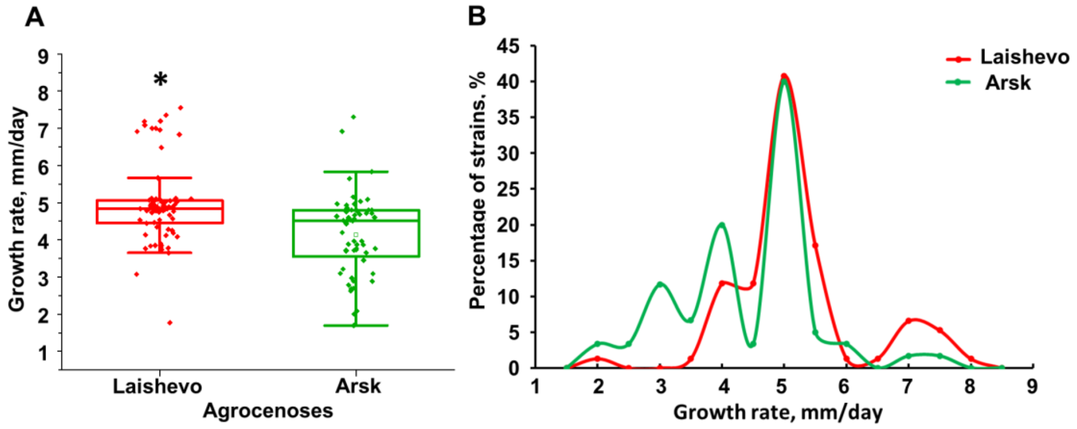

Figure S6. The growth rates (**A**) and the distribution of growth rates (**B**) of *Microdochium nivale* strains from two populations: Laishevo (red) and Arsk (green). Asterisk on the bar indicate significant difference (Mann-Whitney test, $p < 0.05$). Distribution of growth rates was assessed by grouping strains into 15 groups differing in growth rates by 0.5 mm/day.

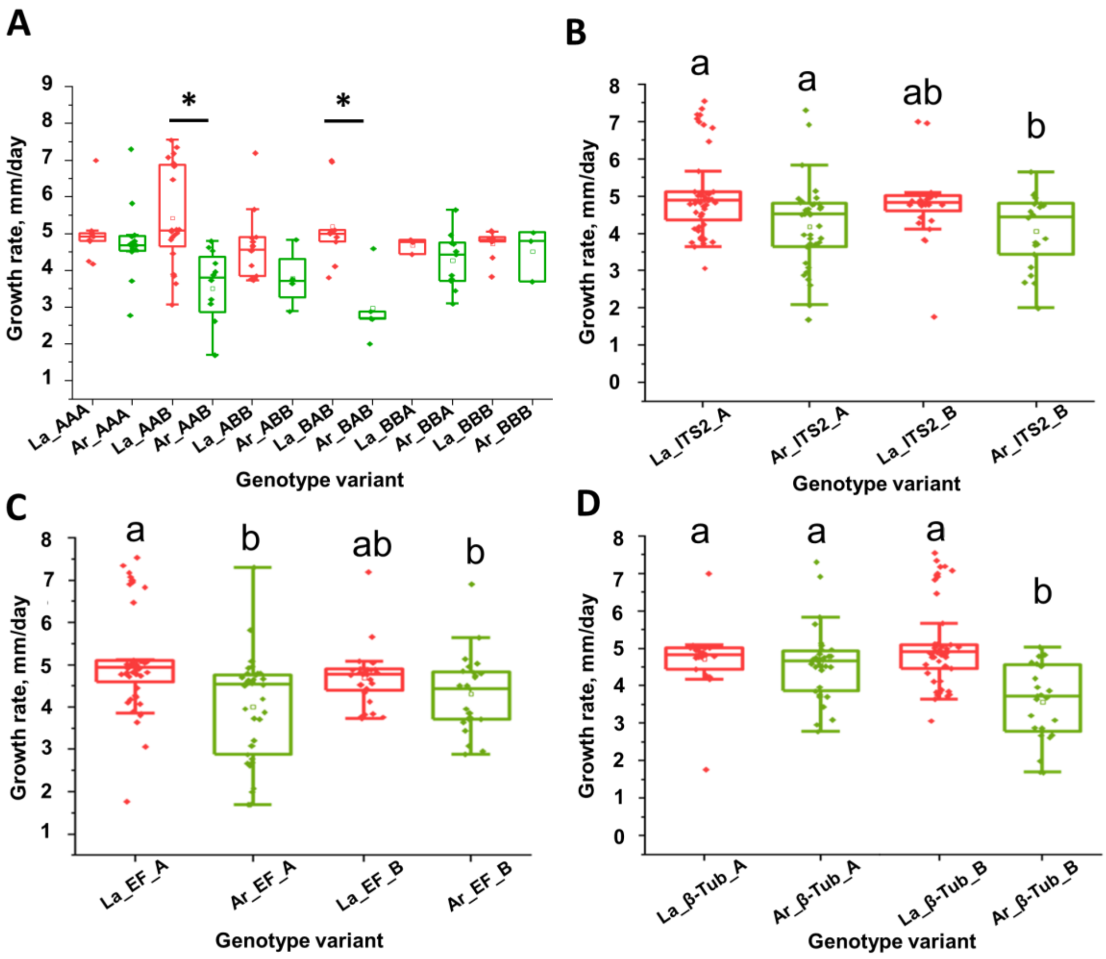

Figure S7. The growth rates of *Microdochium nivale* strains from two populations: Laishevo (La, red) and Arsk (Ar, green). Growth rates were analyzed differentially for strains belonging to six (of the ten revealed) genotypes that were represented in both populations (**A**) and for strains with different sequence variants of the three loci: the internal transcribed spacer 2 (ITS2) (A or B) (**B**), a fragment of the elongation factor 1α gene (EF-1α) (A or B) (**C**), a fragment of the β-tubulin gene (β-Tub) (A, B, or C) (**D**). Genotypes are designated in three letters, where the first letter reflects the ITS2 variant (A/B), the second letter reflects the EF-1α variant (A/B), and the third letter reflects the β-Tub variant (A/B/C). Asterisks in A indicate significant differences in growth rates between similar genotypes originated from different agrocenoses (Mann-Whitney test, p-value < 0.05). Different letters in B-D indicate significant differences (Mann-Whitney test with Bonferroni correction for multiple comparisons, FDR < 0.05).

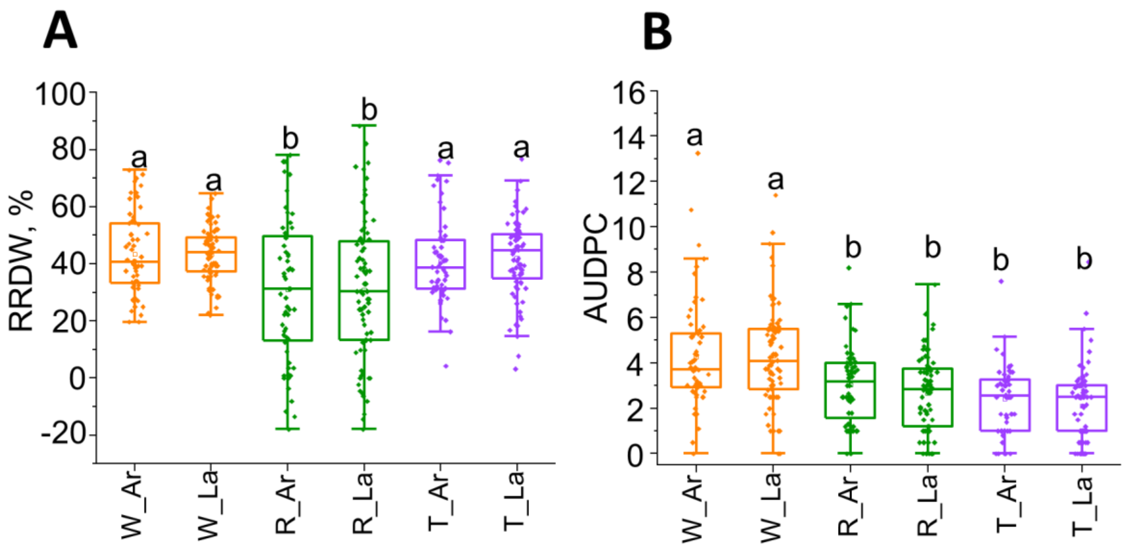

Figure S8. The virulence of *Microdochium nivale* strains from two populations: Arsk (Ar) and Laishevo (La) toward three winter cereal crops (wheat (W), rye (R), and triticale (T)). Virulence was assessed using the whole-plant assay (WPA) (**A**) and expressed as reduced root dry weight (RRDW, %) of infected plants compared to control non-infected plants, as well as using detached leaf assay (DLA) (**B**) and expressed as the area under the disease progress curve (AUDPC). Different letters on the bars indicate significant differences (Mann-Whitney test with Bonferroni correction for multiple comparisons, FDR < 0.05).

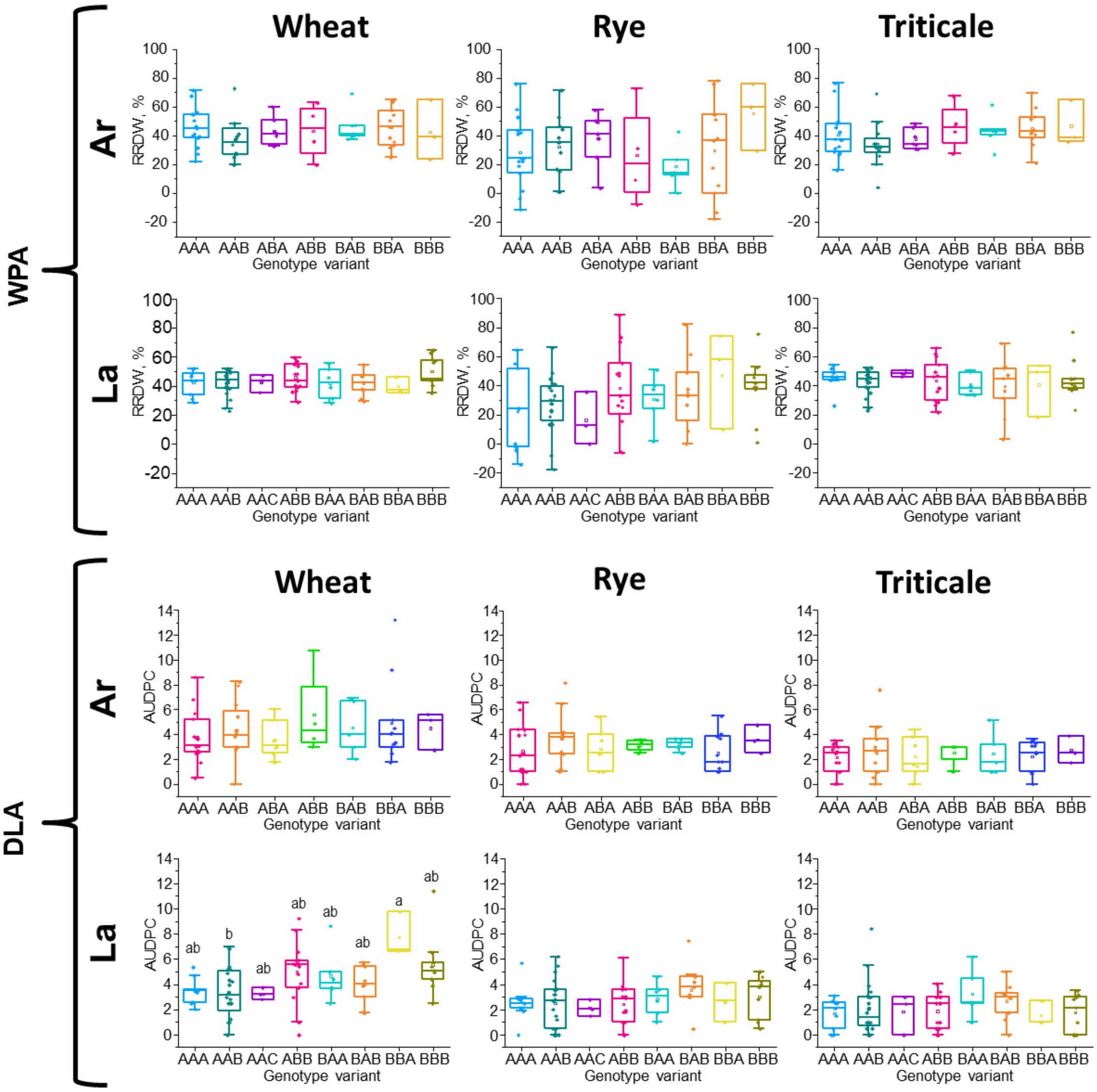

Figure S9. The virulence of *Microdochium nivale* strains from two populations: Arsk (Ar) and Laishevo (La) toward three winter cereal crops (rye, wheat, and triticale). Virulence was analyzed differentially for strains belonging to different genotypes that were represented in either Arsk or Laishevo populations. Virulence was assessed using the whole-plant assay (WPA) and expressed as reduced root dry weight (RRDW, %) of infected plants compared to control non-infected plants, as well as using detached leaf assay (DLA) and expressed as the area under the disease progress curve (AUDPC). Different letters on the bars indicate significant difference (Mann-Whitney test with Bonferroni correction for multiple comparisons, FDR < 0.05).

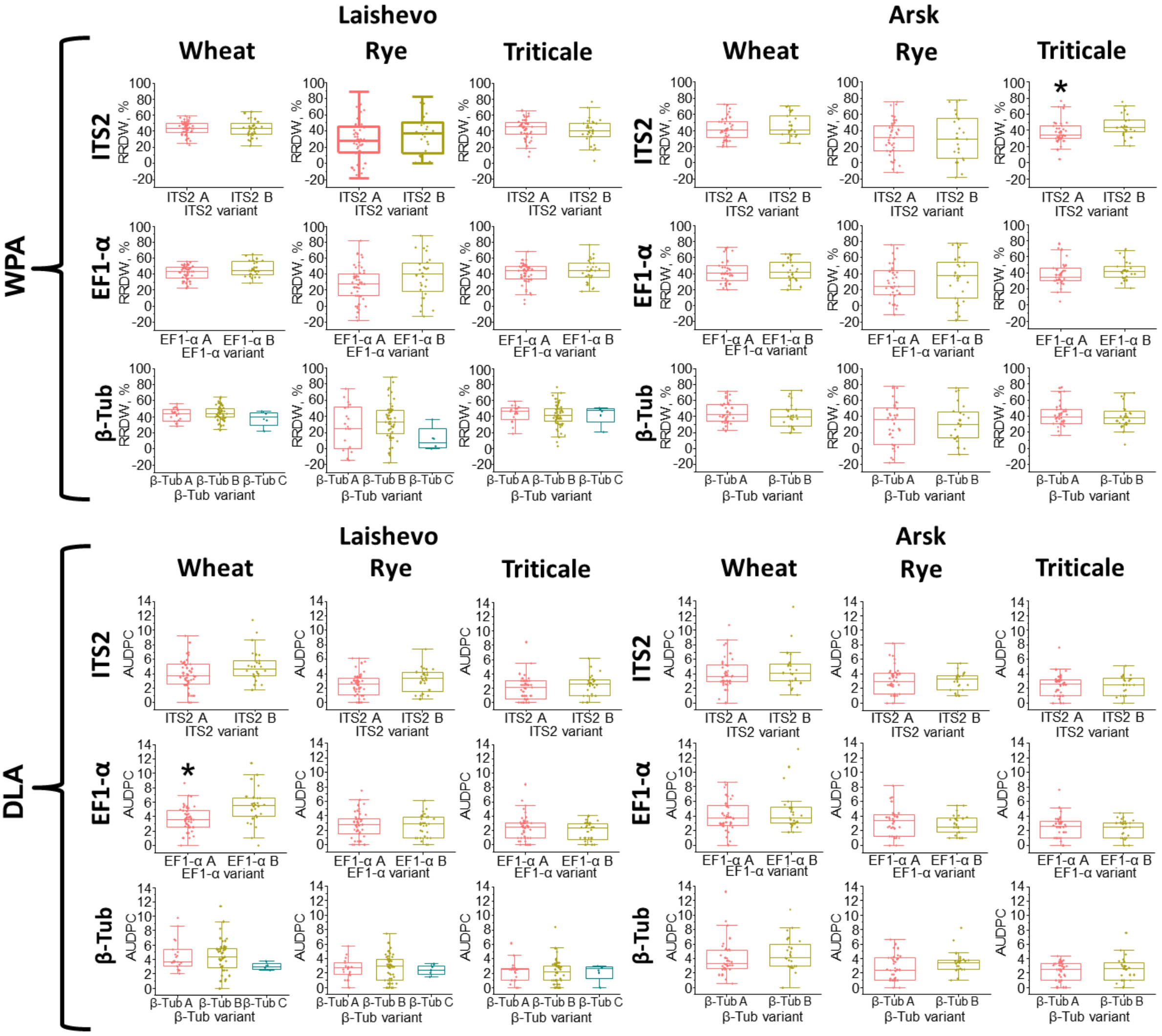

Figure S10. The virulence of *Microdochium nivale* strains from two populations: Arsk (Ar) and Laishevo (La) toward three winter cereal crops (rye, wheat, and triticale). Virulence was analyzed differentially for strains with different sequence variants of the three loci: the internal transcribed spacer 2 (ITS2) (A or B), a fragment of the elongation factor 1α gene (EF-1α) (A or B), a fragment of the β-tubulin gene (β-Tub) (A, B, or C). Virulence was assessed using the whole-plant assay (WPA) and expressed as reduced root dry weight (RRDW, %) of infected plants compared to control non-infected plants, as well as using detached leaf assay (DLA) and expressed as the area under the disease progress curve (AUDPC). Asterisks show significant differences between samples (Mann-Whitney test, $p < 0.05$).
