# Supplementary material for: Intra- and Interpopulation Diversity of the Phytopathogenic Fungi of the Microdochium nivale Species
Source: J Fungi (Basel). 2024 Dec 5;10(12):841. doi: 10.3390/jof10120841 (PMC11678714; doi:10.3390/jof10120841)
Supplement: Supplementary file 1 [file jof-10-00841-s001.zip › Supplementary Table S1.pdf]

Table S1. Comparison of the two agrocenoses considered in the present study

Parameters of locations	Arsk district (Nalasa village)	Laishevo district (Bolshiye Kaban village)
Coordinates (latitude, longitude)	56.113468, 49.774500	55.625164, 49.351334
Average annual temperature, °C *	+ 2.2	+ 4.0
Average annual precipitation, mm *	530	555
Average annual precipitation during the growing season, mm *	356	355
Soil type	sod–podzolic	forest grey
Humus	2.7-2.9	3.2-3.6
Soil bonitet	27.4	28.4
Soil pH	5.9	6.2
Sustainable transition of the daily mean temperature above 0 °C *	10-15 April	24 March – 10 April
Sustainable transition of the daily mean temperature below 0 °C *	Late first to early second decade of November	Second decade of November
Temperature amplitude, °C	84	85
Duration of snow cover, day	183	150
Snow cover height, cm	47	50
Growing season, day	143	147
Absolute minimum temperature, °C	−43… −45	−43… −46
Absolute maximum temperature, °C	+37…+39	+35…+37

* Long-term average data for 1981-2020 recorded at the Hydrometeorology and Environmental Monitoring Department of the Republic of Tatarstan.
